# Anti-Müllerian hormone: a potentially useful biomarker for the diagnosis of canine Sertoli cell tumours

**DOI:** 10.1186/s12917-015-0487-5

**Published:** 2015-07-25

**Authors:** Bodil S. Holst, Ulrika Dreimanis

**Affiliations:** Department of Clinical Sciences, Swedish University of Agricultural Sciences (SLU), PO Box 7054, SE- 750 07 Uppsala, Sweden; Centre for Reproductive Biology in Uppsala, Swedish University of Agricultural Sciences (SLU), PO Box 7054, SE-750 07 Uppsala, Sweden; Department of Small Animals, Helsingborg Referral Animal Hospital, SE-254 66 Helsingborg, Sweden

**Keywords:** Anti-Müllerian hormone, Dog, Neoplasia, Sertoli cell, Testis

## Abstract

**Background:**

Testicular tumours are common in dogs and in many cases do not give rise to clinical signs. In other cases, signs of feminization, hyperpigmentation or alopecia may be observed, most commonly associated with Sertoli cell tumours (SCT). Although these signs are often associated with elevated concentrations of oestradiol, analysis of oestradiol may give inconclusive results due to large variations among individuals. Other biomarkers are therefore needed. Anti-Müllerian hormone (AMH) is expressed by the Sertoli cell. In humans, AMH has been shown to be a specific marker of Sertoli cell origin in gonadal tumours. Using immunohistochemistry, AMH has been shown to be a useful marker of immature and neoplastic Sertoli cells in dogs. The aim of the present study was to evaluate the clinical relevance of AMH analysis in peripheral blood in the diagnostic workup of dogs with suspected testicular tumours.

**Results:**

Blood was collected from 20 dogs with a palpable testicular mass and from 27 healthy controls. Serum was analysed for oestradiol-17β using a RIA and for AMH using an ELISA. The Mann–Whitney U test was used to compare hormone concentrations between different groups.

All control dogs had AMH concentrations ≤ 10 ng/mL, except one outlier that had a concentration of 43 ng/mL. Six dogs with SCT or mixed tumours containing SCT had AMH concentrations higher than 22 ng/mL, significantly higher than AMH concentrations in control dogs (*P* = 0.0004). Concentrations between 10 and 22 ng/mL were found in about half of the dogs with non-neoplastic testicular pathologies or with testicular tumours other than SCTs. Age did not significantly affect concentrations of AMH in the control dogs.

**Conclusion:**

AMH was shown to be a promising biomarker for the diagnosis of Sertoli cell tumours in dogs.

## Background

Testicular tumours are common in dogs, more common than in other domestic species [1, 2]. In a study using the Norwegian Canine Cancer register (all histologically verified tumours submitted from veterinary practices within a defined area in Norway from predominantly intact dogs), 7.1 % of all tumours from male dogs were located in the testis [3]. In studies on unselected adult dogs submitted for routine autopsy, a prevalence of 16 % of testicular tumours was reported in 1962 [2], and 27 % in 2008 [4]. This may indicate an increasing prevalence in dogs, although other factors such as selection bias may contribute. In humans, an increasing incidence of testicular tumours has been described [5]. Poor semen quality, testis cancer, undescended testis and hypospadias have been proposed to be parts of the testicular dysgenesis syndrome (TDS) in humans, that is increasing due to environmental factors such as endocrine-disrupting chemicals [6]. Adverse environmental effects may lead to TDS also in intact dogs [7]. Another cause for a potentially increased prevalence of testicular tumours is genetic factors, as breed differences for both frequency and type of testicular tumours have been described [3]. The prevalence of different tumour types varies between studies, but Sertoli cell tumours (SCT), interstitial (Leydig) cell tumours (ICT), and Seminoma (SEM) are most common [2, 4, 8, 9]. Teratomas are rare [1, 2]. Cryptorchidism is associated with an increased risk of SCT and SEM [10].

Many tumours do not give rise to clinical signs except a palpable testicular mass, and of dogs with seminoma, less than one-third of cases were detectable at clinical examination [2]. Besides a palpable testicular mass, common clinical signs when present are feminization with gynecomastia, hyperpigmentation of the skin and bilateral alopecia. Infertility has been described [11]. These signs are observed mainly in dogs with Sertoli-cell tumours and occasionally also with Leydig cell tumours or seminoma [9, 12, 13]. Testicular tumours are rarely malignant, but when metastases occur they are most commonly found in the iliac lymph nodes and lungs [2, 14]. Bone marrow hypoplasia is a life-threatening condition associated with oestrogen production of tumours, most often SCT, and dogs may develop clinical signs related to bone marrow hypoplasia before the owner has noted other clinical signs related to oestrogen production [15]. Once dogs show clinical signs of bone marrow hypoplasia, the prognosis is guarded [15–17].

In mammals, the earliest specific protein expressed by Sertoli cells is Anti-Müllerian hormone (AMH), also called Müllerian inhibiting substance (MIS), a glycoprotein that belongs to the transforming growth factor (TGF) β family [18]. Sertoli cells produce high concentrations of AMH from the time of testicular differentiation up to puberty, and the main effect is the regression of Müllerian ducts at the initiation of male sex differentiation [19]. In humans, AMH has been shown to be a specific marker of Sertoli cell origin in gonadal tumours [20]. In dogs, AMH was expressed in Sertoli cells from foetuses and pups up to day 45 [21]. Analysis of AMH has increasingly been used in human reproductive endocrinology over the last decade, primarily in the IVF settings, e.g. [22, 23]. In cattle AMH analysis has been useful to predict follicular and ovulatory response to gonadotrophin treatment for embryo transfer [24]. In dogs, AMH has been described to distinguish ovariohysterectomized from intact bitches [25].

A second generation ELISA for analysis of AMH, AMH Gen II (Beckman coulter), has been launched for commercial use. This assay uses the same antibodies as the assay previously used in dogs (Diagnostic Systems Laboratories), but with standards for the calibration curve from another assay (Immunotech) [22], and it measures AMH in human, monkey, bovine, dog and other mammalian species [25, 26]. The present study aimed at evaluating the clinical relevance for analysis of AMH in the diagnostic workup of dogs with alopecia or other clinical signs suspected to be associated with Sertoli cell tumours.

## Methods

### Dogs

In total, 20 dogs admitted for castration because of a palpable testicular mass and 27 healthy control dogs with both testicles descended in the testes and no testicular masses identified by palpation were included in the study. All dogs were five years or older and they were a subpopulation of a previous study [9].

The study was approved by the Local Animal Ethical Committee (M 63–09) and the Swedish Animal Welfare Agency (no. 31-2225/09, 31-2226/09). All owners signed informed consent.

### Histology

Histopathological examination from dogs admitted for castration because of a testicular mass was done as previously described [9]. The testes were cut lengthwise and examined for macroscopic lesions. If lesions were present, tissue samples were taken from those areas and from areas with normal parenchyma. In testes without macroscopic lesions, tissue samples from three different sections of the testes were collected for microscopic evaluation, including cranial, central and caudal parts of the testis and the head of the epididymis. The tissue samples were fixed in 4 % neutral buffered formalin, embedded in paraffin wax, sectioned at 3 to 5 μm and stained with haematoxylin and eosin. The neoplastic changes were classified according to the WHO classification system for tumours of domestic animals [27].

### Hormone analyses

Blood was collected and serum used for analysis of oestradiol-17β using a double antibody oestradiol radioimmunoassay^1^ as previously described [9]. The upper reference limit was set at 40 pmol/L [9]. Serum levels of AMH were analysed using an enzyme-linked immunosorbent assay^2^, according to the manufacturer. Briefly, 20 μL of standards, controls and samples were incubated in an anti-AMH antibody coated microtitration plate. After incubation and washing, anti-AMH biotin conjugate was added to each well. After a second incubation and washing step, streptavidin-horseradish peroxidase was added. After a third incubation and washing step, the substrate, tetramethylbenzidine was added and incubated briefly before adding an acidic stopping solution. The degree of enzymatic turnover of the substrate was determined by dual wavelength absorbance measurement at 450 nm and 620 nm. Samples with concentrations >22 ng/mL were diluted 1:10, 1:100 and 1:1000, if enough serum was available. The intra-assay coefficient of variation (CV) was < 5 % and the inter-assay CV was <15 %.

### Statistical analyses

For statistical analysis, the Mann–Whitney U test using Minitab statistical software was used for comparison between groups. Values are reported as median values and inter-quartile range (IQR). For correlations, the Spearman’s rank order correlation was used. The level of statistical significance was set at *P* < 0.05.

## Results

Dogs with palpable masses were of 15 different breeds and three mixed breed dogs, control dogs were of 17 different breeds and 7 mixed breed. In total, twenty-nine breeds were represented. No breed was represented by more than four dogs. The dogs with testicular masses had a median age (IQR) of 8.9 years (8.0-10.8). The control dogs were significantly (*P* < 0.001) younger, with a median age of 6.6 years (5.9-7.8).

Of the 20 dogs with palpable testicular masses, six had ICT, three had SEM, four had SCTs, three had mixed tumours (MIX), two of which contained SCT (MIX-SCT), and four had non-neoplastic pathologies (NNP). For details, see Table 1.

**Table 1:** Details on dogs with palpable testicular masses

Dog	Breed	Age (years)	Tumour	Size/number of tumours	AMH (ng/mL)	Oestradiol (nmol/L)	Comments
1	Petit Basset Griffon Vendéen	7	ICT	0.7 cm	3	6	
2	Mixed breed	11	MIX: SCT and ICT	Multiple	86	7	
3	Border Terrier	9	SCT	5 cm	>22	24	
4	Norwegian Elkhound	7.7	ICT	1 cm	1.8	13	
5	Medium Poodle	12	ICT	3 cm	5.9	21	
6	Rough Collie	8	SCT	Multiple	472	13	
7	German Shepherd	11	MIX^a^	Multiple 1 cm	1246	9	
8	Giant Schnauzer	8.5	SEM	NA	14.3	12	
9	Golden Retriever	8.8	ICT	NA	1.7	5	
10	Norwich Terrier	9.5	SEM	4 cm	12.3	10	
11	Leonberger	7.5	SEM	NA	4.2	16	
12	Golden Retriever	11.3	MIX: ICT and SEM	10 cm	14.2	20	
13	Mixed breed	8	NNP	NA	11.9	11	Multifocal lymphocytic epididymitis
14	Flat Coated Retriever	8.2	NNP	2 cm	12.0	7	Pyogranulomatous fibrous epididymitis and periorchitis
15	Jack Russell Terrier	11.8	NNP	NA	1.9	8	Hemorrhagic necrosis
16	West Highland White terrier	8	NNP	2 cm	15.6	11	Epididymitis and chronic orhchitis
17	German shepherd	10	ICT	Multiple	19.0	49	
18	Miniature Schnauzer	7	SCT	1.5 cm	>22	26	
19	Fox terrier	9	SCT	5 cm	>22	127	Cryptorchid dog
20	Mixed breed	9.3	ICT	1 cm	12.9	97	One tumour in each testis

^a^Mixed germ cell sex cord tumour from Sertoli cells and germ cells

ICT: Interstitial (Leydig) cell tumour; SCT: Sertoli cell tumour, SEM: Seminoma; MIX: mixed testicular tumours; NNP: Non-neoplastic pathology

The median concentration of AMH in the 27 control dogs was 5.1 ng/mL (3.8-7.1). All control dogs had AMH concentrations ≤ 10 ng/mL, except for one outlier that had a concentration of > 22 ng/mL: 43 ng/mL. This dog was lost to follow-up. Age did not significantly affect concentrations of AMH in the control dogs (r_s_ = 0.2, *P* = 0.2).

**Fig. 1:**
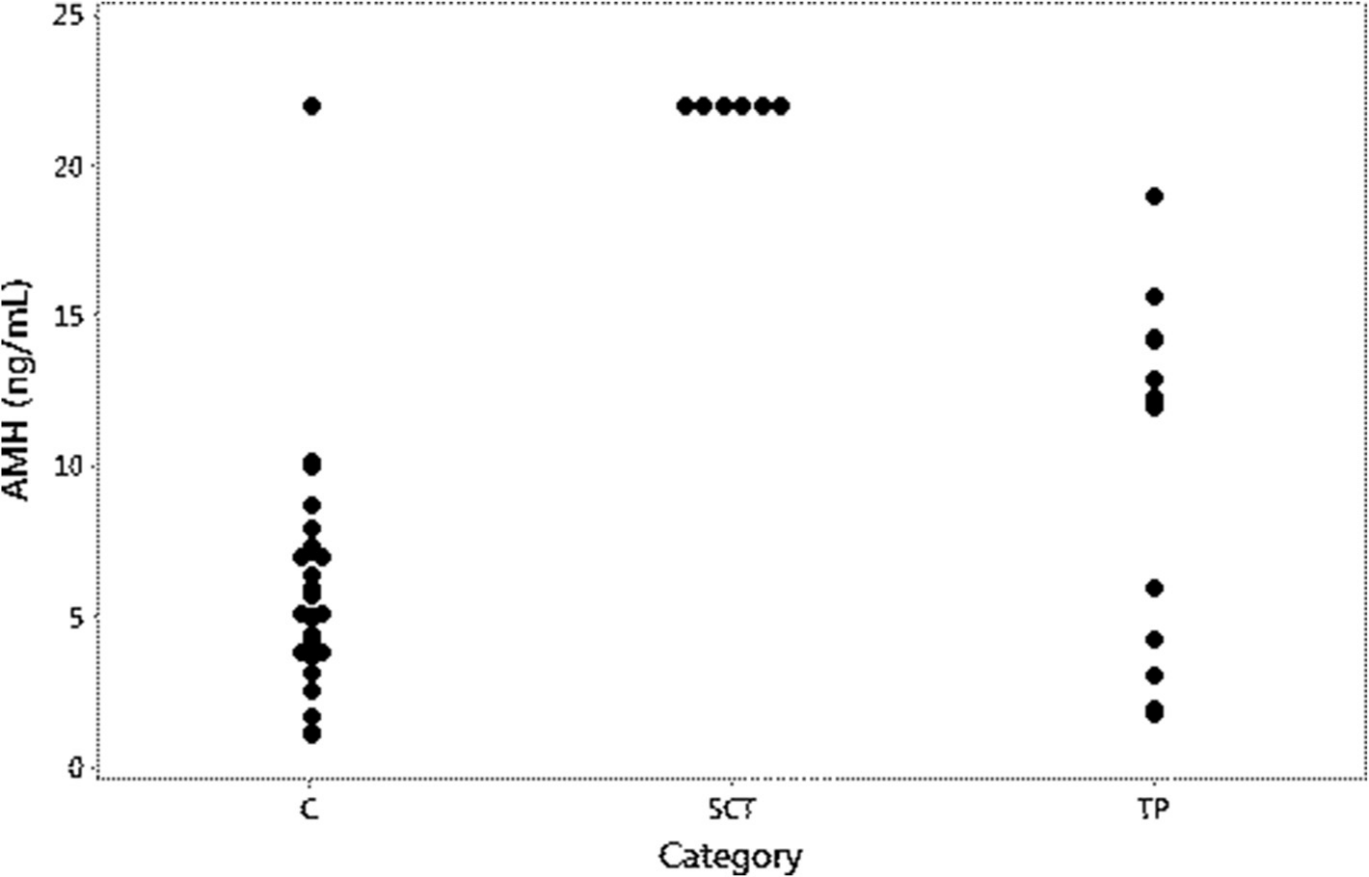
AMH concentrations in control dogs and in dogs with testicular pathologies. C: control dogs, SCT: Dogs with tumours containing neoplastic Sertoli cells; TP: Dog with testicular pathologies not containing neoplastic Sertoli cells. Concentrations of AMH > 22 ng/mL are plotted as 22 ng/mL in the graph

The six dogs with SCT or MIX-SCT had AMH concentrations above the highest standard point. In three of these dogs a further dilution was not possible, and in these dogs final concentrations are reported as 22 ng/mL. The six dogs with SCT or MIX-SCT had significantly higher concentrations of AMH (54.0 ng/mL, 22.0-66.0) than the control dogs (*P* = 0.0004). They also had higher AMH concentrations than the group of 14 dogs with other testicular tumours or NNP (12.0 ng/mL, 2.7-14.2). Of these 14 dogs 8 had AMH concentrations between 10 and 22 ng/mL (2/3 with SEM, 3/4 with NNP, 1/1 with MIX-non SCT, and 2/6 with ICT) (Table 1, Fig. 1). The AMH concentration did not differ significantly between dogs with ICT, SEM, or NNP (Table 2).

**Table 2:** Concentrations AMH and estradiol (median and inter-quartile range, IQR) in control dogs and dogs with testicular pathologies

Classification of dogs	Number of dogs	Median AMH concentration, ng/mL (IQR)	Median estradiol concentration, pmol/L (IQR)
Control dogs	27	5.1 (3.8-7.1)	14.0 (11.0-17.0)
NNP	4	12.0 (4.4-14.7)	9.5 (7.3-11.0)
SEM	3	12.3 (4.2-14.3)	12.0 (10.0-16.0)
ICT	6	4.5 (1.8-14.4)	17.0 (5.8-61.0)
MIX-nonSCT	1	14.2	20.0
SCT + MIX-SCT	6	54 (22–666)	18.5 (8.5-51.3)

NNP: non-neoplastic pathology; SEM: Seminoma; ICT: Interstitial (Leydig) cell tumour; SCT: Sertoli cell tumour, MIX-nonSCT: Mixed testicular tumour not containing Sertoli cell tumour; SCT + MIX-SCT: Sertoli cell tumours and mixed testicular tumours containing SCT

**Fig. 2:**
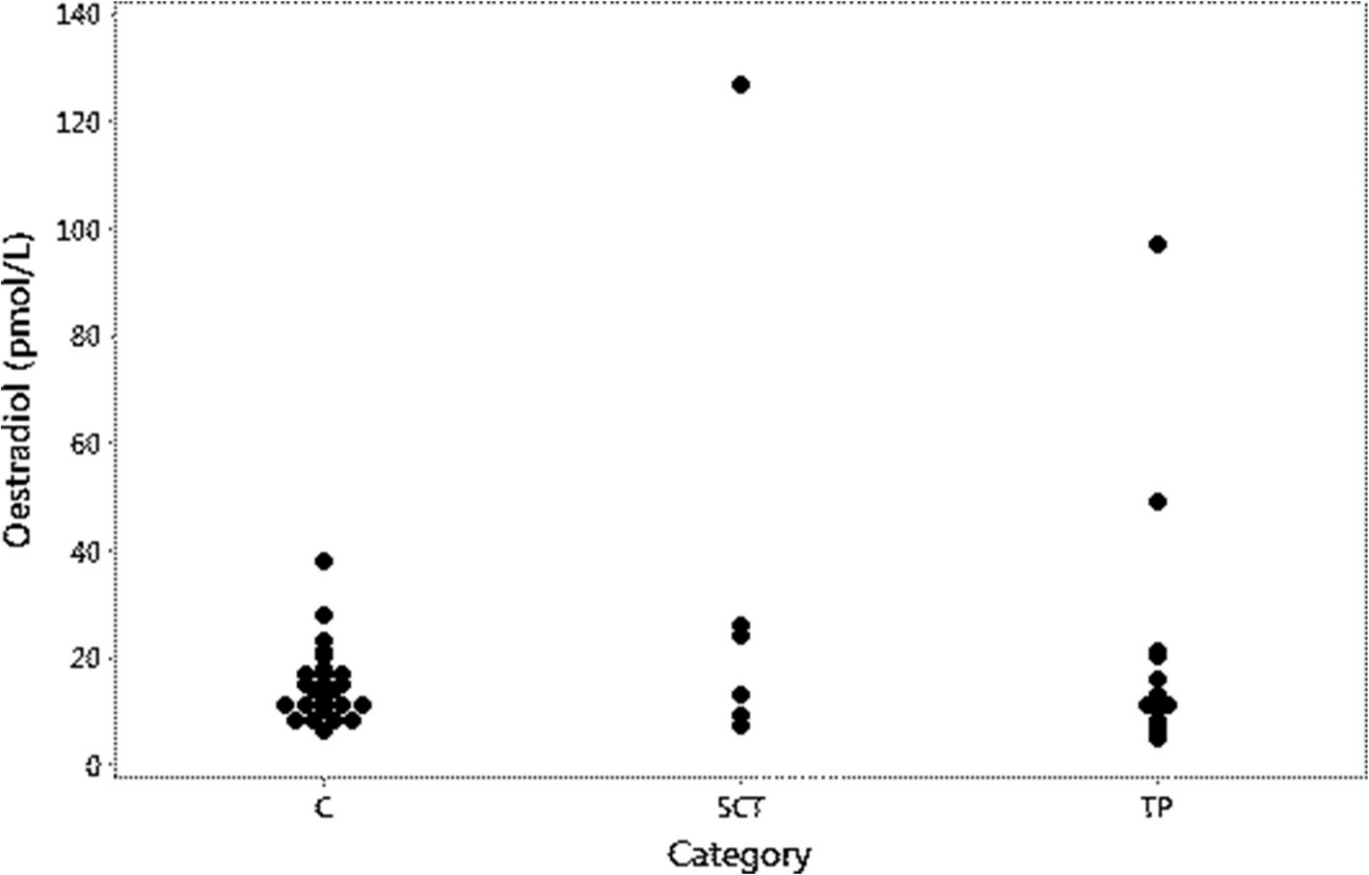
Oestradiol concentrations in control dogs and in dogs with testicular pathologies. C: control dogs, SCT: Dogs with tumours containing neoplastic Sertoli cells; TP: Dog with testicular pathologies not containing neoplastic Sertoli cells

Three dogs had elevated oestradiol concentrations: one dog out of the six with SCT or MIX-SCT and two out of the six dogs with ICT (Table 1, Fig. 2). The median oestradiol concentration was 14.0 pmol/L (11.0-17.0) in the control dogs, and 18.5 pmol/L (8.5-51.3) in dogs with SCT and MIX-SCT. In the group of 14 dogs with other testicular tumours or NNP it was 11.5 pmol/L (7.8-20.2) (Table 2). The differences between the groups were not statistically significant. AMH concentration did not correlate significantly with oestradiol concentration (r_s_ = 0.3, *P* = 0.07).

## Discussion

The present study describes the value of analysing serum concentrations of AMH for diagnosing SCT. All dogs with SCT, including dogs with mixed tumours containing neoplastic Sertoli cells, had high (>22 ng/mL) concentrations of AMH, and in the three dogs in which further dilutions were possible, concentrations were found to be elevated from 4X to 1000X. An elevated serum concentration of AMH associated with SCT has previously been reported in a dog with non-pruritic alopecia [28]. The results of the present study and of that case report are in accordance with a previous study, using immunohistochemistry, reporting that AMH is a useful marker for immature and neoplastic canine Sertoli cells [21].

In a previous study on the canine testis, AMH was not expressed by Leydig cells, spermatogonia, interstitium or the epididymis [21]. In castrated male cats, the AMH concentration has been described to be below the detection limit [29]. In the present study, all control dogs, except for one outlier, had detectable AMH concentrations ≤ 10 pg/mL. AMH concentration did not differ significantly between control dogs and dogs with testicular tumours other than SCT or with NNP, but about half of dogs with these testicular changes had serum concentrations in the interval between 10 and 22 pg/mL. An increased expression of markers of immaturity in Sertoli cells, including AMH, has recently been described in cases of canine testicular atrophy [30]. Such changes may contribute to slightly elevated concentrations of AMH in some dogs with testicular pathologies other than SCT.

The control dogs were checked for palpable testicular masses and were clinically healthy, but no ultrasonography or histopathological examination of the testes was carried out because they were not castrated. This is a limitation of the study because minor testicular lesions cannot be ruled out. Sertoli cell tumours can be small and may not be detectable on a clinical examination, even if they give rise to clinical signs [2, 4, 11]. The range of AMH concentrations in dogs with no histopathological lesions may be narrower than the range found in the control dogs in the present study. The one control dog with a clearly elevated concentration of AMH most likely had a subclinical SCT. Unfortunately, this dog was lost to follow-up.

Another study limitation was that the control dogs were significantly younger than the dogs with testicular pathologies. However, age was not found to significantly affect AMH concentration in the control dogs. In humans, AMH concentrations have been described to decrease with age in older men although with large inter-individual variations [31]. It is thus highly unlikely that the increased AMH concentrations in dogs with testicular pathologies are related to age.

Oestradiol concentrations were high in one out of four dogs with SCT and in two out of six dogs with ICT. Whereas AMH is produced solely by Sertoli cells, increased production of oestradiol can be caused also by other testicular tumours but most commonly by SCTs [9, 12, 13]. In addition, serum oestradiol can be used as a part of an adrenal panel [32] because oestrogens not only are synthesized in the gonads, but also in the adrenals and in other tissues, such as skin and adipose tissue [33]. A substantial variability in oestradiol concentrations has been described both between and within castrated dogs [34]. Clinical signs of feminization may be associated with increased concentrations of oestradiol-17β or, more often, with a decreased testosterone/oestradiol ratio [9, 12]. There are thus several drawbacks with serum oestradiol analysis as a tool to diagnose SCT. To diagnose oestrogen producing tumours in dogs, preputial cytology may be an alternative [9].

There are several clinical implications of the study. In human medicine, AMH analysis has been described as a valuable tool to evaluate gonadal function in paediatric male hypogonadism [35–37] and Sertoli cell origin in gonadal tumours [20]. Testicular tumours that cause alopecia are not always associated with feminisation or increased concentration of oestradiol in dogs [9, 38], and even in cases of bone marrow suppression, clinical signs of feminisation may not be noticed by the owner [15]. In addition, oestradiol concentrations are not always elevated in cases with feminization [12]. Analysis of AMH may thus be useful in the diagnostic workup both of patients with dermatological problem and of patients with bone marrow suppression. Sertoli cells play a crucial role in spermatogenesis, and alterations in Sertoli cell function may lead to impaired spermatogenesis and male infertility. Non-palpable SCT have been associated with infertility in dogs [11]. Because AMH concentrations are increased in dogs with SCT, as shown in the present study, and an elevated expression of AMH has been described in testicular degeneration [30], analysis of AMH is of potential value in the diagnostic workup of dogs with reduced fertility. The one cryptorchid dog in the present study had a SCT, so the effect of cryptorchidism on AMH concentration could not be established. In horses, geldings have serum concentrations of AMH that are at or below the detection limit of the assay, whereas cryptorchid stallions have higher AMH concentrations than stallions with descended testes [39]. In cats, castrated males had AMH concentrations below the detection limit [29]. Analysis of AMH may be useful for determining if a dog is castrated or cryptorchid.

## Conclusions

AMH is a biomarker for canine SCT, and can therefore be of value in the diagnostic work-up of e.g. male dogs with signs of feminisation, hyperpigmentation, alopecia, subfertility, infertility and bone marrow suppression. Further studies will be needed to verify reference intervals, because other testicular pathologies may also induce elevated concentrations of AMH, although generally lower than SCT.

## Abbreviations

AMH: Anti-Müllerian hormone
ICT: Interstitial (Leydig) cell tumour
IQR: Inter-quartile range
MIX: Mixed tumours
MIX-SCT: Mixed tumours containing SCT
NNP: Non-neoplastic pathology
SCT: Sertoli cell tumour
SEM: Seminoma

## Footnotes

1 coat-a-count, Siemens Healthcare Diagnostics

2 AMH Gen II ELISA, Beckman coulter
